# ‘Too much, too little’ – heat wave impact during pregnancy and the need for adaptation measures

**DOI:** 10.1080/16549716.2025.2476277

**Published:** 2025-03-13

**Authors:** Ashish Kc, Masoud Vaezghasemi

**Affiliations:** a School of Public Health and Community Medicine, Institute of Medicine, University of Gothenburg, Gothenburg, Sweden; b Department of Epidemiology and Global Health, Medical Faculty, Umeå University, Umeå, Sweden

**Keywords:** Heat wave, vulnerability among pregnant women, heat adaptation, heat-associated preterm birth, ecological crisis

## Abstract

The balls are rolling for climate change, with increasing vulnerability to women and children related to climate extreme events. Recent evidence has shown that acute exposure to heat wave during pregnancy can be associated with adverse health outcomes in childhood, with the risk being significantly higher among socially disadvantaged population, despite their lack of contribution to global carbon dioxide emissions and the rising global ambient temperature. This unequal impact requires utmost attention to develop tools, establish interdisciplinary teams, and to implement evidence-based interventions for the betterment of women and children in climate-vulnerable populations.

## Paper context

- **Main findings:** Amid the climate crisis, extreme events disproportionately affect vulnerable populations in low- and middle-income countries (LMICs), particularly women and children.
- **Added knowledge:** Climate-related extreme events, such as heat waves, significantly impact vulnerable populations, yet there are insufficient measures in place to reduce the vulnerability of women and children.
- **Global health impact for policy and action:** There is a critical need to establish interdisciplinary teams, generate evidence from existing local data, develop robust tools, and, most importantly, strengthen health systems to mitigate the impacts of climate change.

## What is the context

In June 2024, for two consecutive weeks, the ambient temperature in Chitwan and southern belt of Nepal, bordering India, reached 44-degree centigrade with high humidity [1]. The high ambient temperature in the southern belt has been in rise since last two decades with the number of events increasing year by year but never had witnessed such sustained high temperature [2,3]. Chitwan like other part of Nepal has an area where 30% of socially disadvantaged and poor reside in poor housing conditions with little to no access to water [4]. Like Nepal, in South Asia, one fourth of the global population reside, pregnant women face the dual risk of ecological crisis and sociological stress [5–8], where increasing exposure to heat wave has led to poor birth outcomes such as preterm birth and small for gestational age [9,10].

In our recent study in Nepal, using a secondary analysis of a 2017–2021 perinatal dataset of 74,446 pregnant women from the seven heat-prone districts, the risk of preterm birth among pregnant women who were exposed to non-optimal ambient temperature in comparison to those exposed to optimal ambient temperature was investigated [11]. The perinatal dataset was collected using hospital register for gestational age and obstetric complication and semi-structured interviews with pregnant women on socio-economic status by independent research teams [12]. The ambient temperature was extracted from the global metrological data repository, National Oceanic and Atmospheric Administration (NOAA) [13]. A space–time-stratified case-crossover design was used to assess association of daily number of preterm births to mean daily temperature stratified by socio-economic position adjusted to obstetric complication. The study used distributed lag non-linear models (DLNMs) to estimate the exposure–response function. The result showed that pregnant women who were exposed to ambient temperature >75th centile had preterm birth increases by 29% [11]. The risk of preterm birth increased by 81% when the ambient temperature rose to 90th centile. Importantly, the risk of preterm birth due to high ambient temperature exposure was influenced by social positioning.

This indicates that in comparison to women from advantaged population with higher access to resources, women from disadvantaged populations have more than 80% higher risk of preterm births during heat waves [11]. Furthermore, with the increasing disparity in Nepal and South Asia, the climate extreme events are going to disproportionately affect women from poorer communities [14].

There might be several possible biological pathways for women from poorer socio-economic condition where heat is a mediator for increasing risk of preterm birth. First, these women are at higher risk of poor immunity to infection, leading to ascending infection through cervix, consequently leading to inflammation or damage to fatal membrane and pre-term premature rupture of membranes [15]. Second, women who are in higher psychological and/or physiological distress due to living conditions, exposure of heat further mediates the increase in serum cortisol level. The increase in systemic cortisol might trigger the premature activation of cyclooxygenase-2 (CoX-2) in the placenta and membranes, causing preterm labour and preterm birth [16,17]. These factors can make a heat wave or high temperature event trigger an acute physiological response, such as the release of cortisol, leading to premature delivery [18]. Furthermore, it is also possible that this physiological response can also be caused when the ambient temperature falls below 25th centile [11].

## What is known

In the last 10 years, there have been a number of epidemiological studies from high and middle income settings on the effect of high ambient temperature or heat waves on preterm birth [19–24] and very few from low-income settings [10,25]. A systematic review on the impact of a one-degree temperature increase on preterm birth found that most studies were conducted in high-income settings, highlighting a significant lack of data from low-income regions [26]. Epidemiological and biological evidence suggests that high ambient temperatures affect premature delivery and preterm birth. Nevertheless, the pre-existing high psychological and physiological stress in low resource settings and disadvantaged populations in comparison to high resource settings and advantaged populations might be an effect modifier when pregnant women are exposed to high ambient temperature [27,28]. As we have shown, social conditions and social positionality are key effect modifiers when considering the linkage between high ambient temperature to preterm birth [11]. However, further investigation is needed using eco-spatial datasets on social conditions, ambient temperature, and preterm birth. Employing diverse study designs in various settings will help identify the most vulnerable populations. Additionally, there is a lack of qualitative studies and knowledge on how societies and populations perceive increasing high ambient temperature. This information is valuable for developing interventions to increase awareness and improving health system readiness to manage pregnant women exposed to high ambient temperature. However, evidence shows that too little has been done for too much of the problem.

### How to adopt

As planetary health scientists and funders have focused their resources and attention on investigating the effect of heat waves during pregnancy and postnatally in Africa and Asia, governments now need assessment tools, adaptation measures and health system readiness to address heat wave impact.

To our knowledge, there are no standardized tools to measure population risk perception to heat wave and high ambient temperature [29]. Thus, it is imperative for the research community to develop, validate and standardize tools which can assess the heat wave risk perception among the women during and after pregnancy [30]. The Health Belief Model (HBM) can serve as a framework for developing a tool to assess heat-risk perception among pregnant women, as it has been effectively utilized in various population groups and contexts [31]. This is of vital importance, as health policymakers and managers require standardized heat risk perception measuring tools to better understand adaptation measures. Such tools will provide guidance to policymakers and program managers on necessary interventions to improve risk perception, identify barriers to managing the risk, and understand the benefits of adaptation.

There are different adaptation measures to manage heat wave during pregnancy, such as air conditioning, installing more inside blinds or outside shadings, adding more green or blue areas around the house or on the balcony, using a fan or moving into a cooler house or city [32]. However, the implementing the adaptation measure is contingent upon the resources that families and women can afford [33]. Other adaptation measures such as eating cold food, taking frequent showers and frequent hydrating the body may also be available for women to manage heat waves [33]. Further contextually relevant explorations are need on different effective measure according to geographical terrain, economic conditions, housing conditions and cultural customs.

Based on our current evidence and women’s knowledge and risk perceptions [34], health systems must take proactive steps to prepare women and families for the challenges posed by heatwaves, while ensuring that healthcare systems are adequately equipped to manage the unique needs of pregnant women exposed to high ambient temperatures. At present, many health systems in LMICs lack the necessary infrastructure, training, and resources to effectively address the health impacts of heat stress on pregnant women, highlighting a critical gap in preparedness and response.

## What is next?

The tackling of exposure due to climate events on poor health outcomes which are beyond and outside the realm of health section requires an interdisciplinary approach [35]. Interdisciplinary scientists, managers and implementation team can help to understand and implement the solution. Since, to understand the epidemiology of why heat waves disproportionately affect disadvantaged populations requires medical physiologists, epidemiologists and environment scientists, while to develop and validate tools to assess heat risk perception, social scientists and epidemiologists need to work together. As an example, a medical physiologist would investigate the biological mechanisms of heat events and their pathophysiological effects on pregnant women. An epidemiologist would employ new epidemiological tools to assess exposure and outcomes through mediational analysis. An environmental scientist would provide accurate methods to measure ambient temperature and environmental exposure. Meanwhile, a social scientist would further develop qualitative approaches to explore women’s experiences with heat and facilitate community-based participatory research to involve pregnant women and other stakeholders in the research process, ensuring that their voices and experiences are heard.

There is also a need for further interdisciplinary fellowship programs like, the Swedish Institute of Global Health Transformation (SIGHT) hosted by the Royal Swedish Academy of Sciences in 2017. Through the program 11 mid-career researchers – including the authors of the text – from multiple disciplines were selected across Sweden to address grand global health challenges though the UN agenda 2030 perspective, including climate change and biodiversity loss [36]. Most importantly, what united the fellows was their strong desire to reach across disciplinary boundaries and leverage this diversity towards formulating a fresh interdisciplinary approach to global and planetary health challenges. We, therefore, feel that initiatives such as the SIGHT Fellowship program deserve to be further supported by funding agencies as well as the Swedish universities – in various possible formats – to champion early-career researchers and provide them with the platform, tools, and support needed to tackle global challenges from an interdisciplinary perspective.

Finally, the ongoing ecological crisis and heat waves will have untoward event in pregnant women and children. This will be further exacerbated with the global ambient temperature crossing 1.5 degree Centigrade, causing further biodiversity loss biodegradation, agricultural insecurity and poor health conditions. The breaching of planetary safe boundaries [37], resulting in large-scale environmental degradation and rising ambient temperatures, is now being acutely felt by women and other vulnerable populations in regions such as the southern belts of Nepal. We need interdisciplinary teams, equal South-South and North-South partnerships, effective tools, adaptation measures and health system readiness to prevent the disproportionate impact of high ambient temperature on socially disadvantaged populations and pregnant women. It is crucial to generate more robust evidence by utilizing existing health and climate data, alongside practical examples of adaptation strategies at the community level in LMICs, as outlined in our text. While viewpoints and commentaries can provide valuable perspectives, a stronger emphasis on data-driven research and evidence-based practices will be more impactful in driving meaningful changes. We have too much to do with too little time to spare.
